# Altitude-Related Change in Endotracheal Tube Cuff Pressures in Helicopter EMS

**DOI:** 10.5811/westjem.2017.3.32078

**Published:** 2017-05-15

**Authors:** Stacy N. Weisberg, Jonathan C. McCall, Joseph Tennyson

**Affiliations:** * University of Massachusetts Medical School, Department of Emergency Medicine, Worcester, Massachusetts; † Ochsner West Bank Hospital, Department of Emergency Medicine, Gretna, Louisiana

## Abstract

**Introduction:**

Over-inflation of endotracheal tube (ETT) cuffs has the potential to lead to scarring and stenosis of the trachea.^1^, ^2^,^3^, ^4^ The air inside an ETT cuff is subject to expansion as atmospheric pressure decreases, as happens with an increase in altitude. Emergency medical services helicopters are not pressurized, thereby providing a good environment for studying the effects of altitude changes ETT cuff pressures. This study aims to explore the relationship between altitude and ETT cuff pressures in a helicopter air-medical transport program.

**Methods:**

ETT cuffs were initially inflated in a nonstandardized manner and then adjusted to a pressure of 25 cmH_2_O. The pressure was again measured when the helicopter reached maximum altitude. A final pressure was recorded when the helicopter landed at the receiving facility.

**Results:**

We enrolled 60 subjects in the study. The mean for initial tube cuff pressures was 70 cmH_2_O. Maximum altitude for the program ranged from 1,000–3,000 feet above sea level, with a change in altitude from 800–2,480 feet. Mean cuff pressure at altitude was 36.52 ± 8.56 cmH_2_O. Despite the significant change in cuff pressure at maximum altitude, there was no relationship found between the maximum altitude and the cuff pressures measured.

**Conclusion:**

Our study failed to demonstrate the expected linear relationship between ETT cuff pressures and the maximum altitude achieved during typical air-medical transportation in our system. At altitudes less than 3,000 feet above sea level, the effect of altitude change on ETT pressure is minimal and does not require a change in practice to saline-filled cuffs.

## INTRODUCTION

Air-medical critical care providers are frequently called upon to provide advanced airway management to our critically ill patients. The majority of these cases involve the transport of patients who have been intubated with standard endotracheal tubes (ETT).

ETT cuffs are typically instilled with 10 ml of air. This allows a closed system of ventilation via respirator or bag valve mask (BVM). However, measuring the volume of air instilled frequently does not equate to proper pressure of the ETT cuff on the trachea. It has been previously demonstrated that some ETT cuffs have been over-inflated, defined as pressures in excess of 30 cmH_2_O. This has the potential to lead to ischemia and subsequent scarring and stenosis of the trachea.^1^, ^2^, ^3^, ^4^, ^5^ This occurs because the pressure of the cuff against the tracheal mucosa is greater than the pressure of the capillary beds supplying the blood flow to this structure.^4^,^6^ Studies have shown that this may be more pronounced in hypotensive states, as in septic shock.^7^ There have even been case reports of tracheal rupture related to over-inflation of ETT cuffs.^8^ One study reported rates of tracheal stenosis as high as 22%, of which 1–2% were clinically significant.^9^

The air inside ETT cuffs is subject to the forces of atmospheric pressure, which allows it to expand and contract. This is best illustrated by the application of Boyle’s law, which states that the volume of a given gas relates inversely to its pressure (P1V1 = P2V2). The pressure effects of altitude on cuff volume are predicted by Boyle’s law, which states that a fixed mass of gas will expand as ambient pressure decreases.^10^ If there is no method of venting this expansion, there will be an increase in pressure within any air-filled space, such as in the fixed diameter of the trachea.^11^ Therefore, the air inside of an ETT cuff is subject to expansion as atmospheric pressure decreases, as happens with increase in altitude.

Because emergency medical service (EMS) helicopters are not pressurized, they provide an ideal environment for directly studying the effect of altitude changes on the pressure inside the ETT cuff. Fixed-wing aircraft are pressurized to maintain stable atmospheric pressures of 3,000–8,000 feet above sea level, depending on the aircraft type. Critical care crews operating in these aircraft commonly use saline rather than air to fill patients’ ETT cuffs.^7^ It might follow that this should be a consideration in non-pressurized EMS helicopters as well. This also applies to pediatric patients, as more cuffed ETTs are being used in younger patients.^12^

Our goal in this study was to explore the relationship between altitude and the pressure in ETT cuffs. We hypothesized there would be a significant increase in ETT cuff pressures when at altitudes at which EMS helicopters typically fly and that there would be a relationship between maximum altitude and ETT cuff pressures.

## METHODS

The subjects enrolled in this study were critically ill and were determined to meet the criteria for critical care transport by an outside medical facility or ground EMS service. The patients were all intubated prior to air transport by the referring medical team or by the Life Flight air medical team. The referring medical team was either a hospital or an EMS/ambulance ground crew. We excluded patients intubated with an uncuffed ETT from the study due to the inability to measure pressures. Prisoners were also excluded from the study per institutional review board recommendations.

The ETT cuffs were inflated in a non-standardized manner by the intubating personnel. Using a commercially available device, the Posey Cufflator^TM^ Endotracheal Tube Inflator and Manometer (Posey Company, 5635 Peck Road, Arcadia, CA, USA), an initial ETT cuff pressure was recorded by the Life Flight medical team team prior to air transport. If this reading was found to be greater than 25 cm H_2_O, enough air was removed to bring the pressure below 25 cm H_2_O. If the reading was less than 25 cm H_2_O, the ETT was examined for the presence of an air leak. If an air leak was detected, enough pressure was added to the cuff to eliminate it. Air transportation of the patient was then initiated.

**Population Health Research Capsule**

What do we already know about this issue?

Many endotracheal tube (ETT) cuffs are over-inflated, potentially causing pressure-related tracheal injuries. In a closed system at altitude, the pressure caused by air in an ETT cuff will increase.

What was the research question?

Is there a significant increase in ETT cuff pressures when at altitudes that EMS helicopters typically fly?

What was the major finding of the study?

There is not a linear relationship between ETT cuff pressures and maximum altitude during transports near sea level.

How does this improve population health?

The data support routine monitoring of ETT cuff pressures, as many cuffs were initially over-inflated. However, at altitudes near sea level, there is no need to replace air with saline.

The pilot alerted the crew once the aircraft had reached the maximum planned altitude. At that point the ETT cuff pressure was rechecked. Both the altitude and the cuff pressure were recorded. This was repeated for a total of three measurements at cruising altitude. Upon landing at the destination, the crew again checked and recorded the cuff pressure. Where altitudes were reported as a range, the average was calculated and used for the remainder of the study.

We collected study data via a research form completed by the air medical crew upon transfer of patient care to the receiving facility. The form contained minimal demographic information (age, gender, and diagnosis). The data were entered into a spreadsheet for storage (Microsoft Excel, Microsoft Corporation, Redmond, WA). We analyzed the data using IBM SPSS Statistics (IBM Corp. IBM SPSS Statistics for Windows. Armonk, NY)

No chart review or patient follow up was performed, as such details were not pertinent to the variables being studied. The study was granted approval after University of Massachusetts Institutional Review Board review.

## RESULTS

We enrolled a total of 60 subjects in the study. Subjects ranged in age from 18–90 years old. Thirty-nine (65%) were male (*p* value for the difference was not significant at p=0.27). The majority of patients’ conditions were medical in nature (47 of 60, 78%) with trauma accounting for only 13 of 60 or 22% (*p* value for the difference at p<0.001). The majority of patients were intubated prior to air medical transport arrival (56 of 60, 93%, p<0.001) (Table).

**Figure 1:**
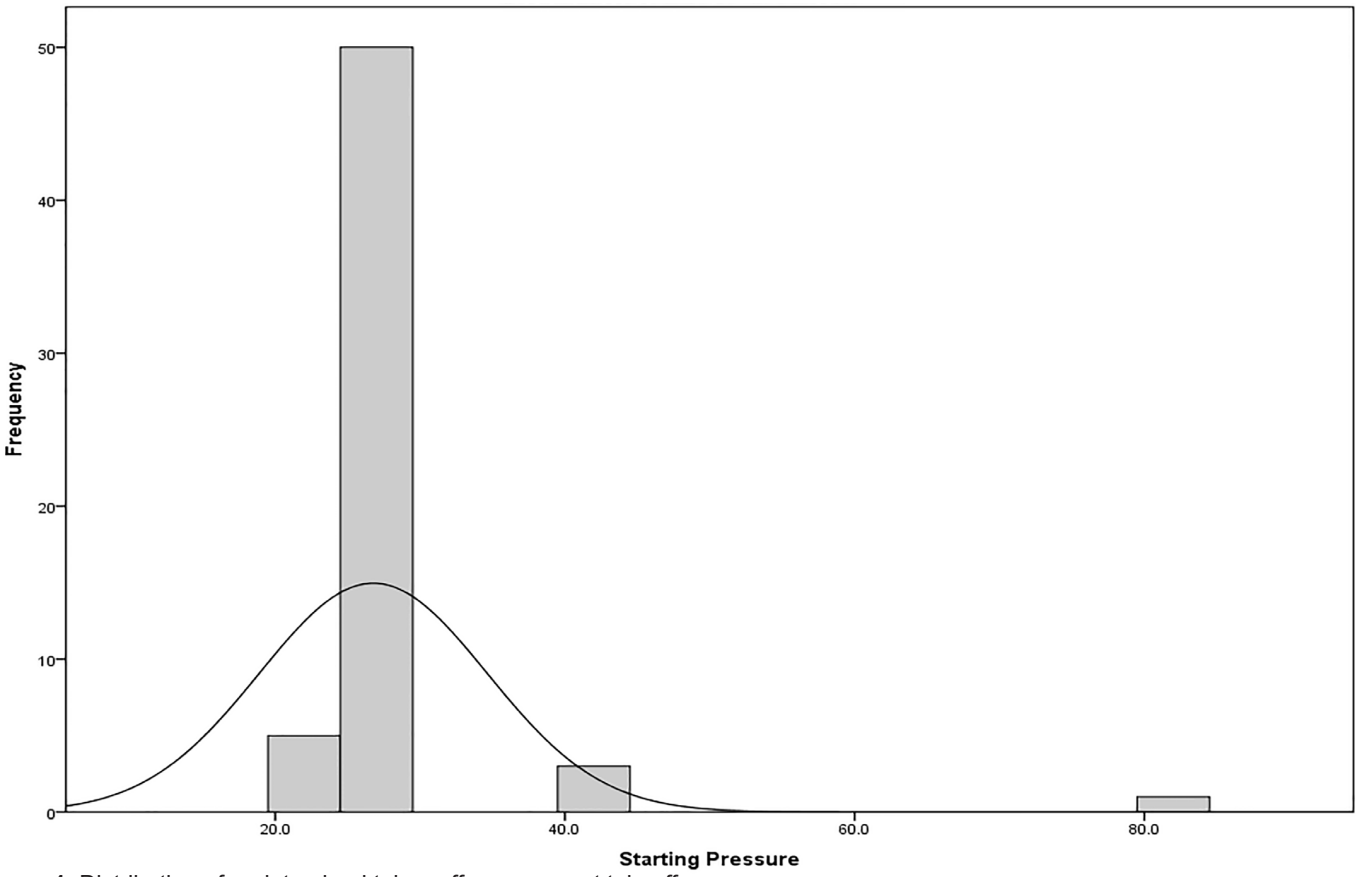
Distribution of endotracheal tube cuff pressures at takeoff.

Initial cuff pressures were measured and recorded for all but one patient and the majorities were well above the recommended pressure. Therefore, air was removed from the balloon to obtain an initial cuff pressure of mean 25.12 cm ± 3.93 cmH_2_O (Figure 1).

The mode for initial cuff pressure for patients intubated by the referral agency was 120 cm H_2_O. Based on an analysis of this subset (56/60 patients) the mean initial cuff pressure measurement was 70 cmH_2_O, 40 cmH_2_O higher than the accepted maximum safe value of 30 cmH_2_O (p<0.0001, 95% confidence interval [CI] for the difference 31–50). This portion of the data is explored in more detail in a separate paper.

In a minimum of cases, the lowering of the initial cuff pressure resulted in a leak of air around the ETT cuff during positive pressure ventilation. The study protocol addressed this eventuality by including a protocol for inflating the cuff to the minimum pressure needed to stop a cuff leak in cases where a cuff leak was noted at normalization. This was an infrequent occurrence (6/60, 10%). The average pressure needed to seal the cuff was 42 ± 23 cm H_2_0.

**Figure 2:**
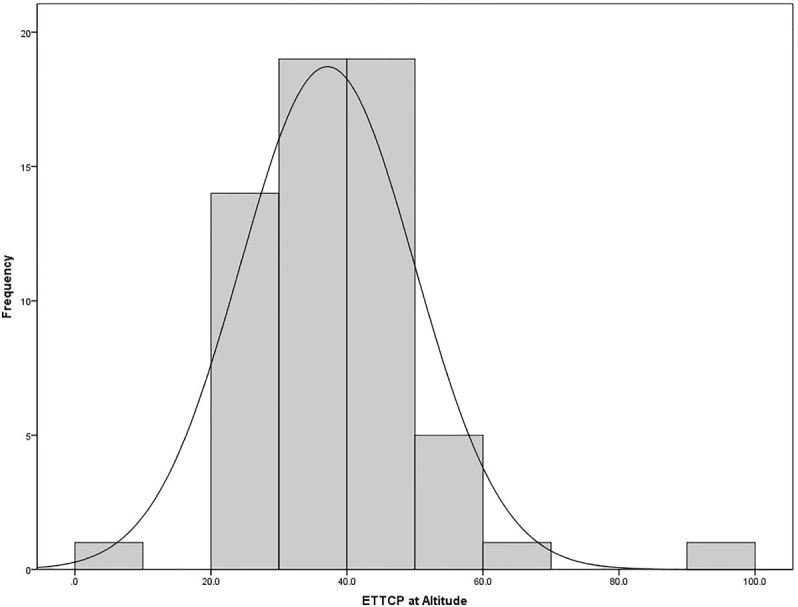
Distribution of endotracheal tube cuff pressures (ETTCP) at altitude.

Maximum altitude measurements were recorded for all subjects and ranged from 1,000–3,000 feet above sea level, mean 1,931 feet. Change in altitude from initial measurement to maximum flight altitude ranged from an increase in 800 to 2,480 feet. The mean increase in altitude was 1,420 ± 392 feet. Cuff pressures at maximum altitude ranged from 22–78 cm of water with a mean cuff pressure of 36.52 ± 8.56 cmH_2_O (Figure 2).

The result of the *t* test for paired means comparing cuff pressure at departure and at maximum altitude is significant (t_49_ = −10.53, p < 0.001).

**Figure 3:**
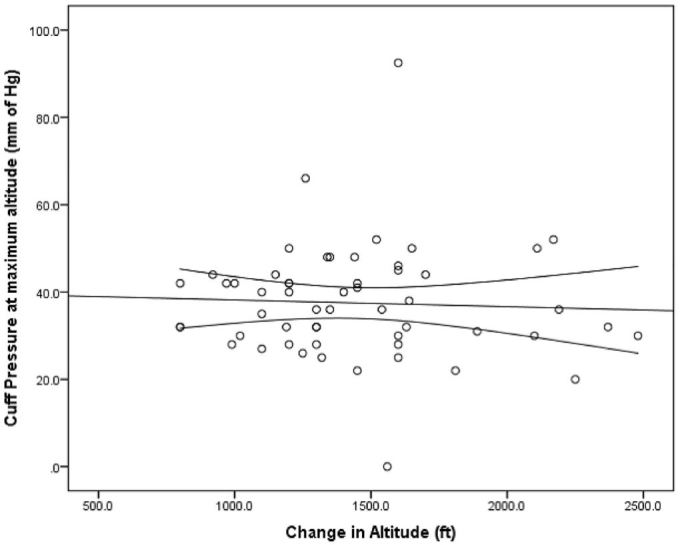
Comparison between cuff pressure at maximum altitude and change in altitude.

Despite the significant change in cuff pressure at maximum altitude, there was no relationship found between the maximum altitude and the cuff pressures measured (slope = −0.033, p= 0.803, R^2^=0.001). Taking cabin temperature or provider into account as possibly affecting cuff pressure did not change the results (slope= +0.011, p= 0.947, R^2^=0.009) (Figure 3).

The mean change in pressure from starting to cruising altitude was 10.8 ± 10.9 cmH_2_O (95% CI [8–14]). The median change was 10 cmH_2_O (IQR [3–18]). When at altitude, 41 (68%) had pressures >30 cmH_2_O. Four patients (7%) had pressures > 50 cmH_2_O. One patient (2%) had a pressure >80 cmH_2_O.

## DISCUSSION

Our study failed to demonstrate the expected linear relationship between ETT cuff pressures and the maximum altitude achieved during typical air-medical transportation in our system. Controlling for other variables, including cabin temperature and ventilator settings, did not change the lack of relationship. These findings contradict the findings in previous studies, which suggested that tube pressure increases at altitude, leading to recommendations to measure tube cuff pressures and inflate cuffs with saline instead of air.^1^

Despite the lack of a reproducible relationship at maximum altitude or increase in altitude, our results do demonstrate an increase in the pressures from those established prior to initiation of flight. Pressures increased on average almost 11 cmH_2_O with 77% of cases exceeding the maximum recommended pressure of 30 cmH_2_O while at altitude.

Both animal and human studies have demonstrated evidence of harm from increased ETT cuff pressure.^2^, ^3^, ^4^ Seegobin performed tracheoscopy on patients whose ETT cuff pressure had exceeded 40 cmH_2_O and found decreased blood flow evidenced by mucosal blanching.^4^

Complications reported in humans associated with increased ETT cuff pressure have ranged from the less severe realm of hoarseness, sore throat, and minor hemoptysis^13^ to the more severe of post extubation stridor^14^ and tracheal stenosis.^9^, ^15^ There are even reports of tracheal rupture.^8^,^16^ An association between elevated ETT cuff pressure and tracheal stenosis was demonstrated by Kastanos in his 1983 paper.^15^

One reason for the lack of relationship may be due to the altitudes of this flight program. The helicopter flew at a maximum altitude of 3,000 feet above sea level, with an average altitude of 1,931 feet and an average increase in altitude of 1,420 feet. The studies reporting clinically significant changes in tube cuff pressure reported these results at altitudes of at least 3,000 feet.^1^, ^17^ In this study, the mean altitude was only 1,931 feet with only one air-medical mission reporting a maximum altitude of 3,000 feet above sea level.

While this study is only from one flight program, the findings should be generalizable to other programs flying at or near sea level. These data support the routine monitoring of ETT cuff pressure during flight, but do not suggest the need to replace air with saline at altitudes near sea level. We would encourage programs that typically fly above 3,000 feet to monitor their tube cuff pressures for potential increased pressure at altitude.

## LIMITATIONS

This study does have several limitations. First, we obtained all data from a single air-medical transport program. While we may assume that the results are generalizable to other programs operating at similar altitudes, it is possible that there are confounders specific to this program or the transport crews.

This study only contained 60 subjects. While initial calculations suggested that this would be a sufficient number to detect a relationship between altitude and ETT cuff pressures, it is possible that the sample size was insufficient to detect this relationship.

Lastly, this air-medical program operates at altitudes relatively close to sea level. Most other studies examining this relationship studied programs that operated at higher altitudes. This limits the generalizability of our findings across the air medical industry.

## CONCLUSION

We found no clear relationship between change in altitude and change in endotracheal tube cuff pressures in our cohort of missions flown at altitudes at or less than 3,000 feet above mean sea level. At these altitudes, the effect of altitude change is minimal and does not require a change in practice to saline-filled cuffs. The data do suggest the need for routine monitoring of the pressures during flight. Due to the frequently significantly elevated cuff pressures at the time of patient contact, services should adopt the practice of routinely measuring and normalizing the pressures.

## Figures and Tables

**Table:** Demographics of subjects enrolled in a study of the effect of altitude on endotracheal tube cuff pressure.

Characteristics	Result	Significance
Age (years), mean (95% CI)	56 (51–61)	
Minimum age	18	
Maximum age	90	
Gender, n (%)		P=0.27
Male	39 (65)	
Female	21 (36)	
Nature of case, n (%)		P<0.001
Trauma	13 (22)	
Medical	47 (78)	
ETT size		
Mode	8.0	
Minimum ETT size	6.0	
Maximum ETT size	8.5	
Intubated by air medical crew, n (%)		P<0.001
Yes	4 (7)	
No	56 (93)	

*ETT*, endotracheal tube.
